# Comprehensive Transcriptomic and Epitranscriptomic Profiling of Hepatitis B Virus Transcripts in Two Hepatocellular Carcinoma Cell Lines

**DOI:** 10.3390/genes16121397

**Published:** 2025-11-21

**Authors:** Qinan Zhang, Bohan Zhang, Lei Wang, Yongjian Liu, Jingwan Han, Lei Jia, Hanping Li, Xiaolin Wang, Jingyun Li, Changyuan Yu, Lin Li

**Affiliations:** 1 College of Life Science and Technology, Beijing University of Chemical Technology, Beijing 100029, China; 2023201308@buct.edu.cn (Q.Z.); wanglei@buct.edu.cn (L.W.); 2 Department of Virology, Beijing Institute of Microbiology and Epidemiology, Beijing 100071, China; zbhforjob@163.com (B.Z.); yongjian325@sina.com (Y.L.); hanjingwan@outlook.com (J.H.); 15001193408@163.com (L.J.); hanpingline@163.com (H.L.); woodsxl@163.com (X.W.); lijyjk@163.com (J.L.)

**Keywords:** hepatitis B virus, transcriptome, direct RNA sequencing, alternative splicing, polyadenylation, RNA modifications

## Abstract

**Background/Objectives**: Despite extensive research on hepatitis B virus (HBV), its post-transcriptional regulatory mechanisms remain incompletely characterized, particularly regarding epitranscriptomic modifications. This study aims to systematically profile the transcriptomic complexity and RNA modification landscape of HBV in hepatocellular carcinoma models. **Methods**: We transfected PLC/PRF/5 and Huh7 cells with the HBV 1.3-mer WT replicon plasmid, followed by qPCR measurement of viral load. Total nucleic acids extracted from transfected cells underwent nanopore direct RNA sequencing. The complete HBV transcriptome was then analyzed in two established hepatocellular carcinoma cell lines (PLC/PRF/5 and Huh7), with alternative splicing, polyadenylation, and RNA modifications identified through comprehensive bioinformatics analysis. **Results**: Our analysis revealed substantial transcriptomic diversity, identifying 34 distinct splice variants—including 14 previously unreported isoforms—with cell-type-specific expression patterns. Additionally, we detected 30 high-confidence RNA modification sites across HBV transcripts, with 93% (28 sites) conserved between both cellular environments. Notably, we observed significant intercellular heterogeneity in poly(A) tail length distributions. **Conclusions**: A comparison of the post-transcriptional processing modifications of HBV in PLC/PRF/5 and Huh7 cells reveals that the former may be better able to mimic the immune evasion mechanisms of chronic HBV infection. In contrast, the longer poly(A) tails present in Huh7 cells facilitate efficient replication, rendering these cells more amenable to the study of HBV transcription and replication mechanisms. These findings comprehensively elucidate the post-transcriptional regulatory mechanisms of hepatitis B virus in different hepatocellular carcinoma cell lines, establishing a critical benchmark for selecting appropriate experimental models in virology research. The identified transcriptomic features may provide new insights for developing antiviral strategies targeting the viral epigenome.

## 1. Introduction

RNA processing and modification encompasses key steps such as alternative splicing [1], epigenetic modification [2], and poly(A) tail formation [3], which have been shown to play an important regulatory role in the replication process of various viral infections and exhibit diverse functional characteristics [4,5]. As the main causative agent of chronic hepatitis B and hepatocellular carcinoma (HCC), HBV undergoes series post-transcriptional processing modifications, while the precise and complex mechanisms remain unclear [6].

**Figure 1:**
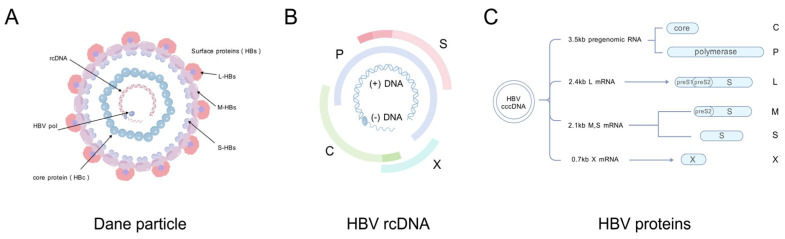
Schematic of HBV virion structure, genome, and transcription. (**A**) Infectious HBV viral particles (Dane particles); (**B**) HBV genomic rcDNA and encoded open reading frames (C, P, S, X); (**C**) HBV transcription–translation machinery (created with BioGDP.com [13]).

The discovery of the hepatitis B virus (HBV) in 1965 led to the recognition of HBV infection as a global public health issue [7]. Different hepatocellular carcinoma cell lines are used for developing therapeutics to resist infection of HBV subsequently [8,9]. Numerous studies have already clarified that the HBV genome is a relaxed circular DNA (rcDNA) molecule approximately 3.2 kilobases (kb) long and encodes four overlapping open reading frames (ORFs): C, P, S, and X [10]. After infection, the rcDNA of HBV genome is then converted to covalently closed circular DNA (cccDNA), which can be transcribed into RNAs of various lengths, primarily 3.5 kb, 2.4 kb, 2.1 kb, and 0.7 kb [11]. Finally, 3.5 kb RNAs are translated into C and P proteins, 2.4 kb RNAs are translated into L-HB, 2.1 kb RNAs are translated into two surface antigens (M-HB and S-HB), and the 0.7 kb RNA are translated into HBx [12] (Figure 1). As a crucial step of HBV replication in cells, the transcription of the HBV genome within cells and the series of regulatory mechanisms during transcription have rarely been studied, and the role of post-transcriptional processing and modification in HBV replication remains unclear as well.

Currently, studies on post-transcriptional processing modifications of the Hepatitis B virus (HBV) have identified over 20 HBV splicing variants, and they speculate that it may help regulate multiple viral and cellular processes in the course of HBV-related liver disease [14,15,16]. In addition, epitranscriptomic analysis have shown that multiple types of epigenetic modifications play an important role in HBV replication. For example, m^6^A modification plays a multifaceted role in HBV replication by regulating HBV RNA stability, nucleocapsid assembly, nuclear export of viral RNA, and HBx expression [17,18]; m^5^C modification has been validated that it is not only essential for Aly/REF export factor recognition to promote viral mRNA export and HBx translation, but also necessary for inhibiting RIG-I binding and suppressing interferon-β production [19]. Pseudouridine (Ψ) modification is another RNA epimodification type widely used in mRNA therapy. Research indicates that a single instance of pseudouridylation may also potentially alter key interactions between viral and host mechanisms in SARS-CoV-2 [20,21]. The formation and length of poly(A) tail show direct impact to RNA stability, for the reason why the process of 3′ adenylation of RNA is one of the most important parts of post-transcriptional modification [22]. Series studies revealed that 3′ adenylation of primary RNA plays an essential role during the maturation and translation of mRNA, while the formation of poly(A) tail of HBV RNA have not been intensively studied yet.

In this study, to investigate the post-transcriptional processing modifications of HBV in various hepatocellular carcinoma cell lines, we employed Nanopore direct RNA sequencing (DRS) technology [23,24], which provides a new solution that can directly focus on individual RNA molecules without PCR amplification and overcome read length limitations since its appearance to sequence HBV transcriptome and then gain insight to HBV transcriptomic and epitranscriptomic features as well [25,26,27]. Our study provides arguments to the post-transcriptional processing modifications of HBV in different hepatocellular carcinoma cell lines and their differences, and further explores the key roles of these regulatory mechanisms throughout the viral life cycle, which is of significant importance for understanding viral pathogenic mechanisms, developing novel antiviral drugs, and optimizing treatment strategies.

## 2. Materials and Methods

### 2.1. Cell Culture and Transfection

The cell lines used in this study were obtained from commercial sources and maintained in our laboratory. PLC/PRF/5 cells were purchased from BeNa Culture Collection (BNCC, Beijing, China), and Huh7 cells were acquired from Procell Life Science & Technology Co., Ltd. (Wuhan, China). Both cell lines were cultured in DMEM medium (Gibco, Thermo Fisher Scientific, Waltham, MA, USA) supplemented with penicillin and streptomycin (Gibco), along with 10% fetal bovine serum (Gibco), under conditions of 37 °C and 5% CO_2_.

For transfection, cells were first seeded in T75 cell culture flasks. When ~90% confluent, they were digested with 0.25% trypsin-EDTA solution (Gibco) and re-plated in 6 cm dishes (1.5 × 10^6^ cells/dish). Upon reaching ~80% confluence, cells were subjected to transfection. The HBV 1.3-mer WT replicon plasmid was purchased from Miaoling Biologicals (Wuhan, China) and transfected with Lipofectamine 2000 reagent (Invitrogen, Thermo Fisher Scientific Inc., Waltham, MA, USA) according to the manufacturer’s instructions.

### 2.2. Extraction of HBV Virion RNA

We used a kit (Takara, Takara Bio Inc., Kusatsu, Shiga, Japan) to extract RNA. We separated the total RNA from the cell sediment. We used the buffer provided in the kit (RL + DTT) to break the cells down, and then followed the instructions of the manufacturer. Finally, we washed the total RNA off using 100 μL of water without a nuclear acid. We measured the total RNA concentration in the sample using the Qubit RNA HS Assay kit (Invitrogen, Carlsbad, CA, USA).

### 2.3. HBV Viral Load Detection

The total sample volume was brought to 200 µL, after which the cellular RNA was extracted using the extraction reagent supplied with the Nucleic Acid Test Kit (Sansure Biotech Inc., Changsha, Hunan, China). The viral load of HBV was then detected by qPCR using the HBV Nucleic Acid Test Kit.

### 2.4. Nanopore Direct RNA Sequencing (DRS-Seq)

For nanopore sequencing of HBV-transfected cells, libraries were prepared using 1000 ng total RNA or 300 ng poly(A) tail RNA according to the manufacturer’s instructions (Oxford Nanopore DRS protocol, SQK-RNA004, Oxford Nanopore Technologies, Oxford, UK). Libraries from all samples were quantified using the Qubit fluorometer DNA HS assay (Invitrogen) and loaded onto a FLO-MIN106D flow cell (R10.4) and then run for 72 h for sequencing on the MinION device (Oxford Nanopore Technologies, Oxford, UK).

### 2.5. In Vitro Transcription

HBV fragments were PCR amplified from the HBV 1.3-mer WT replicon plasmid using designed specific primers (Supplementary Materials, Table S1), PrimeSTAR Max DNA Polymerase (Takara, Kusatsu, Shiga, Japan). The purification process was then initiated using the Promega Wizard SV Gel and PCR Purification System (Promega Corporation, Madison, WI, USA), followed by in vitro transcription using the Vazyme T7 High Yield Transcription Kit (Vazyme Biotech Co., Ltd., Nanjing, China). Subsequently, the transcription purification stage was executed employing the Invitrogen MEGAclear™ Transcription Purification RNA purification was then performed using the Invitrogen MEGAclear™ Transcription Purification Kit (Invitrogen).

### 2.6. DRS Data Preprocessing

Using Dorado (v0.8.1) software, raw electrical signal Pod5 files generated by nanopore direct sequencing were converted into BAM files and their quality assessed. Sequencing reads were directly mapped to the HBV genome using minimap2 (v2.17) with preset splicing parameters. Subsequent filtering, visualization, and sorting operations were performed using Samtools (v1.15.1).

### 2.7. The Splicing Events Analysis of HBV Transcriptome

Classified BAM files were used as input for splice site analysis using megadepth software (v1.2.0) (http://github.com/ChristopherWilks/megadepth, accessed on 9 August 2025). Potential SD and SA site reads were filtered by determining the start and end positions of exons from the BAM file analysis. If the location of potential sites was unknown, only sites associated with typical GT-AG splice site pairs were annotated. Gene Structure Display Server (GSDS, V2.0) was used for genomics data visualization. Gene structure diagrams of the candidate genes were generated using the GSDS 2.0 online tool (http://gsds.gao-lab.org, accessed on 15 October 2025) [28]. The genomic DNA sequences and their corresponding coding sequences (CDS) were submitted to the server to generate the visualizations.

### 2.8. Poly (A) Tail Length Analysis of HBV Transcriptome

Poly(A) tail length detection was performed using Oxford Nanopore Dorado (v0.8.1). The tail interrupt length (default 10 nt) was set to exclude the interference of non-adenylate sequences in the tail interrupt region. The basecalling model was selected in ultra-high precision mode (sup) to enhance the signal segmentation accuracy. The final Poly(A) tail lengths were extracted from the tl tags of the BAM files and summarized statistically by a custom script.

### 2.9. RNA Modification Data Analysis of HBV Transcriptome

The analysis of RNA modification was conducted utilizing the Dorado basecaller (v0.8.1), which incorporates an integrated modification base detection function. Direct RNA sequencing data were analyzed using a dorado basecaller—modified-bases sup,m^6^A_DRACH, m^5^C, pseU/path/to/pod5_files > calls. bam, which simultaneously performs ultra-high-precision basecalls (sup model) and m^6^A_DRACH (context optimization for DRACH-themed motifs), 5-methylcytosine (m^6^A), and 5-methylcytosine (m^5^C). The base probabilities of modifications were extracted from BAM MM/ML markers using modkit (v0.4.3). These were then filtered using a ≥20 probability threshold, and the m^6^A site was further restricted to typical DRACH motifs validated by bedtools nuc (D = A/G/U, R = A/G, H = A/C/U). Finally, the predictions were background corrected against in vitro transcriptional controls.

### 2.10. Statistical Analysis

In GraphPad Prism 8.0.2, the F-test is first performed to assess homogeneity of variance. If *p* < 0.05, the *t*-test with Welch’s correction is used; otherwise, the conventional *t*-test is applied. A *p*-value < 0.05 was considered statistically significant, while a *p*-value < 0.01 was deemed highly significant.

## 3. Results

### 3.1. Direct RNA Sequencing of HBV Plasmid Transfected Cells

**Figure 2:**
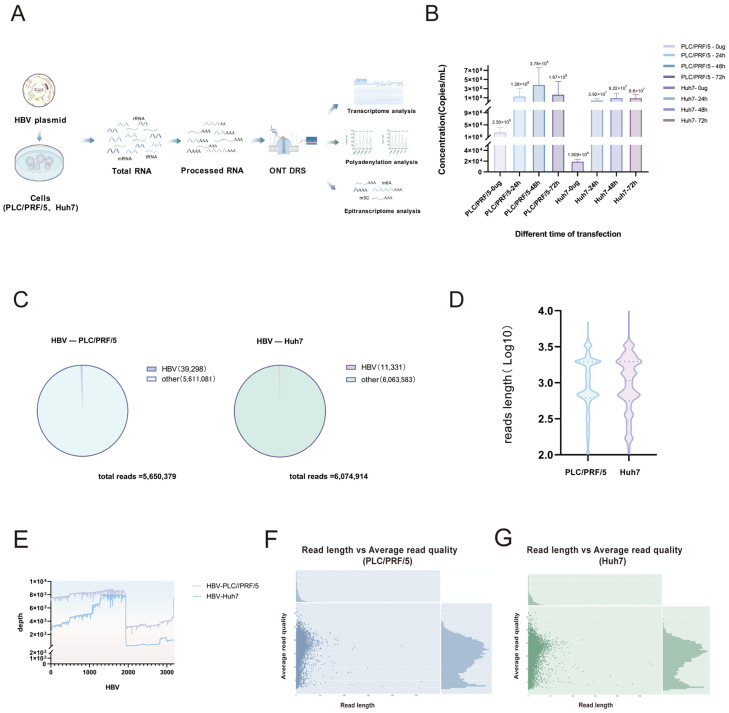
The statistics and features of nanopore direct RNA sequencing data. (**A**) Experimental flowchart (created with BioGDP.com [13], accessed on 17 June 2025). (**B**) Viral load of HBV plasmid transfected PLC/PRF/5 and Huh7 cells. (**C**) Read counts for total reads of cells transfected with HBV plasmid. (**D**) Read length distribution of HBV sequencing data. (**E**) Genomic coverage of HBV sequencing data. (**F**,**G**) Comparative quality values for HBV sequencing data.

To investigate whether there are differences in the processing and modification states of HBV transcripts, we performed nanopore direct RNA sequencing on hepatocellular carcinoma cell lines PLC/PRF/5 and Huh7 transfected with HBV expression vectors (Figure 2A). To obtain comprehensive and optimal sequencing data of the HBV RNA transcriptome, we measured the viral load of cells transfected with different concentrations of HBV plasmids at different time points. The results showed that in both PLC/PRF/5 and Huh7 cells, following transfection with 8 µg of plasmid DNA in 300 µL of Opti-MEM, the viral load reached its peak 48 h post-transfection (Figure 2B).

Therefore, we extracted total RNA from the highest viral load samples of Huh7 and PLC/PRF/5 cells transfected with HBV expression vector (plasmid HBV1.3) followed by nanopore DRS. The total number of reads obtained in HBV-transfected of PLC/PRF/5 cells was 5,650,380, among which 39,298 reads could be aligned to the HBV reference genome sequence. As for transfected Huh7 cells, the total reads were 6,063,583, of which 11,331 reads were found comparable to the reference genome sequence of HBV comparison (Figure 2C). These data indicated that HBV plasmid transfection efficiency was better in PLC/PRF/5 cells than in Huh7 cells. In PLC/PRF/5 and Huh7 cells that had been infected with HBV plasmids, nanopore sequencing read lengths were found to primarily range from 1000 to 3000 nucleotides (Log10 = 3.0–3.5), which is consistent with the theoretical length range of HBV transcripts. Huh7 cells demonstrate a more extensive read length distribution, accompanied by a notable secondary peak of short read lengths (Log10 ≈ 2.8), indicating heightened HBV transcript heterogeneity in comparison to PLC/PRF/5 cells (Figure 2D). Due to the circular structure of the HBV negative strand, 94.81% of HBV transcripts in PLC/PRF/5 cells terminate at the classic HBV transcription termination site (TES, 1930–1950 nt). In Huh7 cells, 92.28% of HBV transcripts terminate at the classical HBV transcription termination site, consistent with the previous literature [15]. Due to significant differences in the total read numbers of the HBV transcriptome between PLC/PRF/5 and Huh7, the coverage depth of HBV transcripts in PLC/PRF/5 is higher than that in Huh7 (Figure 2E). The sequencing read lengths of both cell lines are concentrated in the Q10–Q25 range (average error rate of 0.3–1%), which meets the nanopore standard sequencing quality criteria (typically Q10–Q30). This validates the reliability of the data and supports the reliability of subsequent analyses (Figure 2F,G). These results suggested that the quality and reliability of the raw data of HBV plasmid-transfected PLC/PRF/5 cells and Huh7 cell sequencing were sufficient for subsequent analyses.

### 3.2. Alternative Splicing Event Analysis of Different Hepatocellular Carcinoma Cell Lines

**Figure 3:**
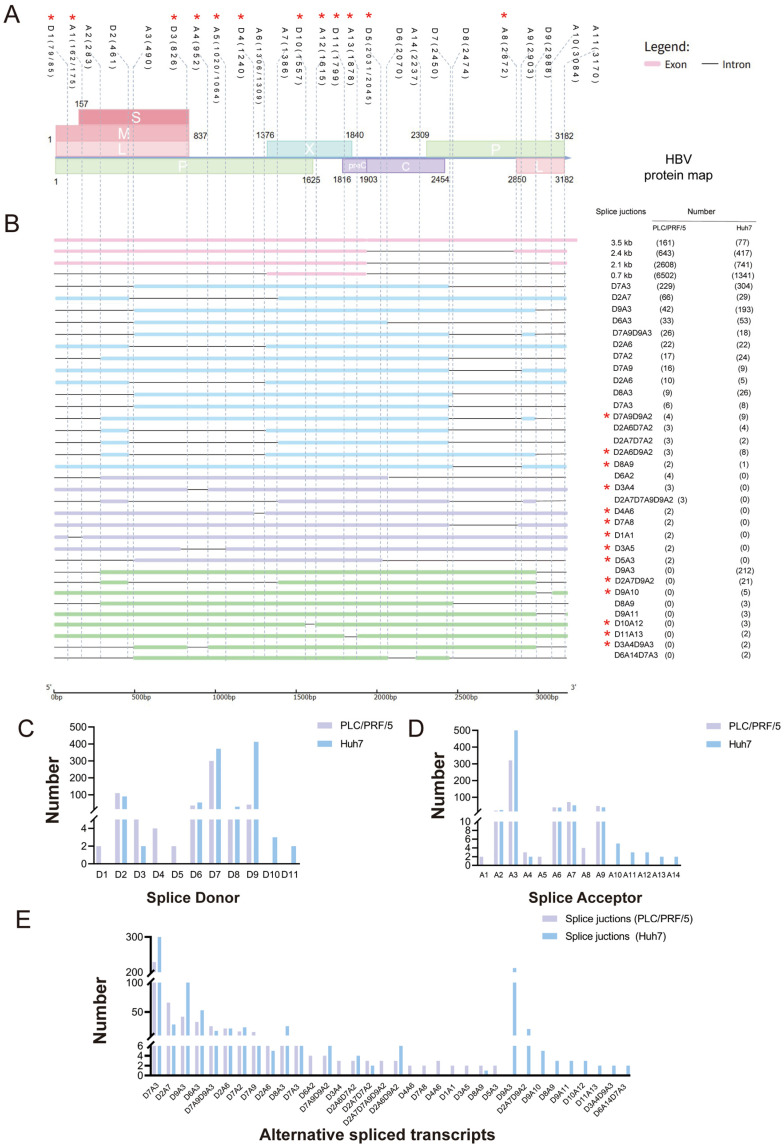
Schematic diagram of hepatitis B virus transcripts. (**A**) Color coding for HBV open reading frames (ORFs) is applied as follows: L denotes light pink, M denotes pink, S denotes dark pink, P denotes green, X denotes blue, and C denotes purple. SD and SA sites should be grouped in a manner conducive to coding. SD and SA sites and their corresponding positions are marked in black. Red asterisks indicate SD and SA sites not yet reported. (**B**) Pink transcripts indicate classic transcripts. Blue transcripts refer to splice variants that are detected in both PLC/PRF/5 and Huh7 cells following transfection with the HBV plasmid. Purple transcripts refer to splice variants that are only present in PLC/PRF/5 cells, but not in Huh7 cells. Green transcripts refer to splice variants that are only present in Huh7 cells, but not in PLC/PRF/5 cells. Red asterisks mark splice variants not previously reported. The number in parentheses indicates the number of splice variants. (**C**) Number of HBV splicing variants calculated based on SD sites classification. (**D**) Number of HBV splicing receptors calculated based on SA sites classification. (**E**) Number of reads for isoforms obtained from HBV plasmid transfected PLC/PRF/5, Huh7 cells.

Based on the DRS data of HBV in the two cell lines, the HBV transcriptomic features were analyzed (Figure 3). As a result, the four canonical HBV transcripts were firstly detected and 0.7 kb transcript was the most detected one in both PLC/PRF/5 cells and Huh 7 cells, which were both sequenced more than 1000 times. In addition, the read number of four canonical HBV transcripts detected in PLC/PRF/5 cells was significantly higher than in Huh7 cells. Except for canonical transcripts, there are other transcripts produced by RNA alternative splicing after HBV infection, thus the similarities and differences in other HBV transcription productions in two cell lines were investigated subsequently. To facilitate description, we consulted definitions of alternative splicing events for viruses such as HIV-1 from former studies [29], identifying the donor and acceptor sites where alternative splicing occurs in the detected HBV transcripts, and thereby naming the transcript types identified through sequencing. As a result, we successfully identified 11 potential splice donor (SD) sites and 13 splice acceptor (SA) sites in the hepatitis B virus (HBV) transcripts, among which nine splicing sites (4 donor site and 5 acceptor site) have not been reported before (Figure 3A). It is worth noting that, some splice sites (D1, D3, D4, D5, D8, D10, D11, A1, A2, A4, A5, A8, and A10–A14) were used less frequently, they were detected in PLC/PRF/5 and Huh7 cells (Figure 3C,D). Moreover, earlier studies have documented the presence of sites D8, A2, A10, A11, and A14, thus substantiating the reliability of this sequencing method for the detection of low-abundance transcripts.

On the other hand, a total of 34 HBV RNA alternative splicing variants were detected in the two cell lines, including 20 previously reported variants [14,30] and 14 newly discovered ones (Figure 3B). Among all alternative splicing isomers, 16 were identified in both PLC/PRF/5 and Huh7 cell lines. Similarly to the splicing events reported before, 2447/489 (splicing editing occurred between 2447 nt and 489 nt) was also found to be the main splicing variant of hepatitis B virus (HBV) [15], namely the aforementioned D7A3, accounting for the majority of spliced transcripts in this study. Additionally, a number of eight HBV splicing variants (D6A2, D3A4, D2A7D7A9D9A2, D4A6, D7A8, D1A1, D3A5, D5A3) were specifically detected in PLC/PRF/5 cells, while a total of nine HBV splicing variants (D9A3, D2A7D9A2, D9A10, D8A9, D9A11, D10A12, D11A13, D3A4D9A3, D6A14D7A3) were only detected in Huh7 cells (Figure 2B). Of particular note was that the D9A3 splicing variants in the Huh7 cell sequencing data, accounting for 22.43% of the total splicing variants, was not found in the PLC/PRF/5 cell sequencing data. Previous studies have demonstrated that HpZ/P′, generated through alternative splicing of viral pre-genomic RNA, inhibits HBV replication and gene expression [31]. Therefore, our newly identified alternative splicing variants may be closely associated with viral replication and transcriptional regulation, and their functions require further validation. Additionally, by comparing the proportion of alternative splicing variants relative to total HBV-associated reads in two hepatocellular carcinoma cell lines (PLC/PRF/5: 1.29% and Huh7: 6.83%), we observed that the proportion of alternative splicing in Huh7 cells was higher than that in PLC/PRF/5. This may be due to the integration of the HBV genome in PLC/PRF/5 cells, and the literature has shown that the methylation intensity of integrated HBV DNA is highly negatively correlated with its transcription level [32], whereas Huh7 cells do not harbor an integrated HBV genome. Taken together, the results supported the notion that investigating HBV alternative splicing across different hepatocellular carcinoma cell lines may reveal complex regulatory mechanisms that provide insights into multiple viral and cellular processes involved in modulating the progression of HBV-associated liver disease, and the results from different hepatocellular carcinoma cell lines suggest that the Huh7 cell line may be more suitable for studies on HBV alternative splicing.

### 3.3. Adenylation Analysis of HBV Transcripts

**Figure 4:**
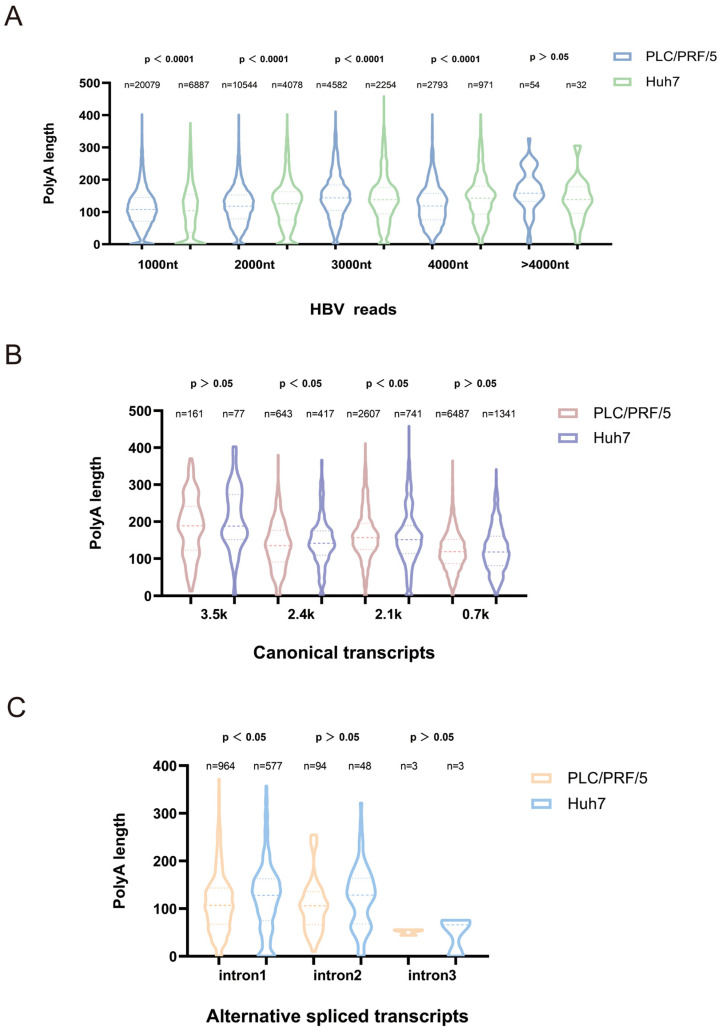
Adenylation analysis profile of hepatitis B virus transcripts. (**A**) HBV plasmid transfected PLC/PRF/5 cells, Huh7 cells—HBV related reads with different lengths of poly(A) tails (Poly(A) tail distributions for 0–1000 nt, 1000–2000 nt, 2000–3000 nt, 3000–4000 nt, >4000 nt, respectively, and n is the number of reads). (**B**) HBV plasmid transfection of PLC/PRF/5 cells, Huh7 cells—Poly(A) tail plot of HBV classical transcripts (Poly(A) tail distributions for 3.5 k, 2.4 k, 2.1 k, and 0.7 k transcripts, respectively, and n is the number of reads). (**C**) HBV plasmid transfection of PLC/PRF/5 cells, Huh7 cells—Poly(A) tail plot of HBV variable shear transcripts (Poly(A) tail distributions of intron1, 2, and 3 variable shear transcripts, respectively, and n is the number of reads).

Previous studies showed that the poly(A) tail of viral transcripts may play a significant role in viral translation, decay, and replication [5]. To investigate the degree of adenylation of HBV transcripts, the present study analyzed the poly(A) length of HBV transcripts in HBV plasmid-transfected PLC/PRF/5 and Huh7 cells using the Dorado tool (Figure 4). Across all HBV transcripts, the mean poly(A) tail length was comparable between PLC/PRF/5 and Huh7 cells (118 nt; Figure 4A). However, stratified analysis by transcript size revealed cell-type-specific differences: for transcripts < 4000 nt, both cell lines exhibited significant differences in polyadenylate length distribution (*p* < 0.001); while for transcripts > 4000 nt, PLC/PRF/5 cells exhibited a bimodal distribution at approximately 150 nt and 250 nt, whereas Huh7 cells showed peaks at approximately 150 nt and 300 nt (Figure 4A). These data indicate that different cell lines exhibit distinct regulation of polyadenylation length for long HBV transcripts, potentially affecting viral RNA stability and genomic integrity.

To further resolve adenylation dynamics, the poly(A) tails length of canonical HBV transcripts (3.5 kb, 2.4 kb, 2.1 kb, 0.7 kb) were analyzed subsequently. The mean length of poly(A) tails of 3.5 k, 2.4 k, and 0.7 k transcripts in the sequencing data of PLC/PRF/5 cells were all shorter than Huh7 cells, while the mean values of 2.1 k classical transcripts were 163 nt in PLC/PRF/5 and 156 nt in Huh7, and for transcripts of 2.4 k and 2.1 k, the polyadenylate length distribution differed significantly between the two cell lines (*p* < 0.05) (Figure 3B). It has been found that the short poly(A) tails of viral RNA may represent senescent RNAs that are susceptible to decay [9], indicating that the PLC/PRF/5 cells were in a much later stage of infection status under the same experimental conditions of 48 h of plasmid transfection.

We next characterized poly(A) tails in HBV splicing variants stratified by intron number (Figure 3C). The mean value of the shear variants with 1 intron in the sequencing data of PLC/PRF/5 cells was 113 nt, and that of Huh7 cells was 123 nt; the mean value of the shear variants with 2 introns in the sequencing data of PLC/PRF/5 cells was 106 nt, and that of Huh7 cells was 124 nt; and the median value of the shear variants with 3 introns in PLC/PRF/5 cells containing 3 intronic shear variants had a median of 51 nt and Huh7 cells had 47 nt. Furthermore, for splicing variants containing a single intron, the polyadenylation length distributions differed significantly between the two cell lines (*p* < 0.05) (Figure 3C). Consistent with the canonical transcripts, most alternative splicing transcripts in Huh7 cells exhibit longer polyadenylation tails than those in PLC/PRF/5 cells, suggesting that PLC/PRF/5 cells may represent a later stage of infection. These results may be due to HBV integration in PLC/PRF/5 cells can influence the expression of genes near the integration site through multiple mechanisms. Their transcription is affected by host epigenetic silencing (such as H3K27me3), leading to reduced efficiency in recognizing the poly(A) signal and resulting in shorter tails [33]. Collectively, these data demonstrate cell-type-specific regulation of poly(A) tail length in HBV transcripts, with PLC/PRF/5 cells exhibiting globally shorter tails in long transcripts and splicing variants. This differential adenylation may contribute to transcript stability control and viral replication dynamics in distinct hepatocyte backgrounds.

### 3.4. Modification Site Analysis of HBV Transcripts in Different Hepatocellular Carcinoma Cell Lines

**Figure 5:**
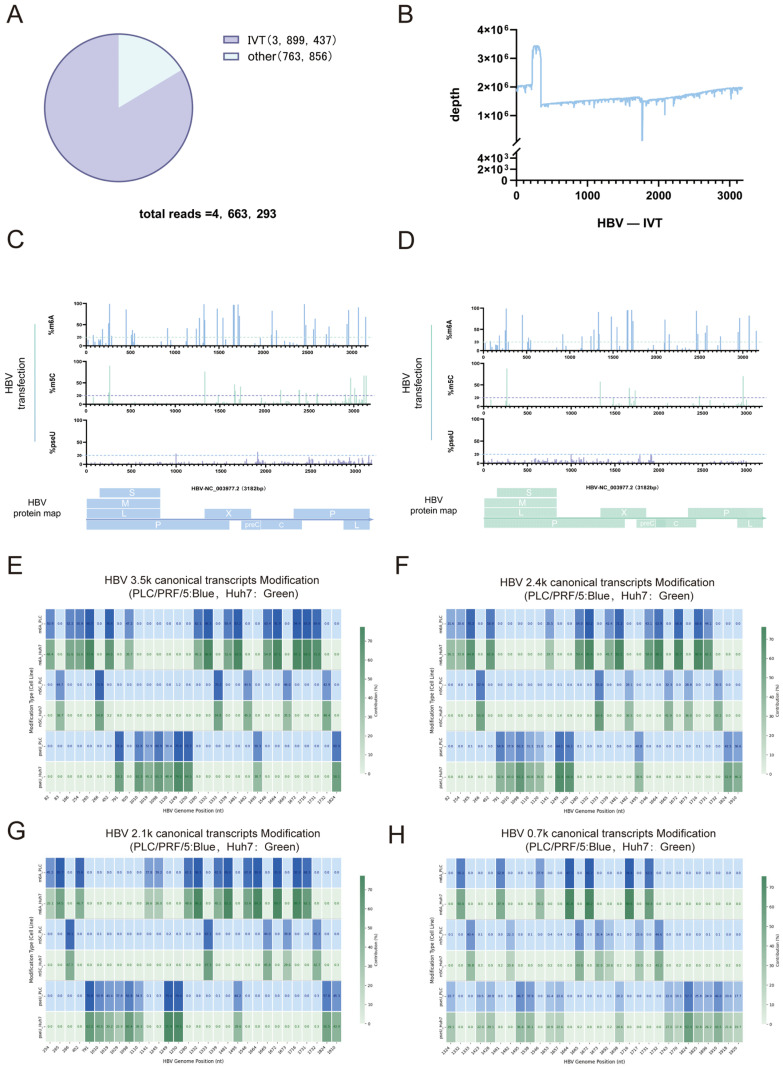
Sequencing data for HBV modification map. (**A**) Count of sequencing reads for in vitro transcribed RNA. (**B**) Genome coverage of IVT RNA sequencing data. (**C**) Sequencing data for HBV plasmid transfected PLC/PRF/5 cells—modification map. (**D**) Sequencing data for HBV plasmid transfected Huh7 cells—modification map. (**E**–**H**) HBV 3.5 k, 2.4 k, 2.1 k, 0.7 k canonical transcripts modification map (PLC/PRF/5: blue, Huh7: green). The numbers in the figure represent the modification rate. We have selected the top thirty sites ranked by modification rate.

As an important component of epitranscriptomics, RNA modifications significantly determine RNA fate, which further affects various biological processes and cellular phenotypes [34]. In this study, the RNA epigenetic modification status of HBV transcripts in PLC/PRF/5 cells and Huh7 cells was analyzed using Dorado Modikit tool. To ensure accuracy of modification site calling, in vitro transcribed (IVT) HBV RNA (via T7 promoter-driven transcription) was used as a modification-negative control and subjected to parallel direct RNA sequencing. The HBV mapping rates and genome coverage indicated the sequencing data were suitable for downstream modification analysis as shown in Figure 5A,B.

Potential RNA modification sites were then identified by comparing electrical signal differences between cellular HBV transcripts and IVT controls (unmodified) (Table 1, Table 2, Table 3, Table 4, Table 5 and Table 6). In total, 30 and 28 modification sites were detected in PLC/PRF/5 and Huh7 cells, respectively (Figure 5C,D). Among them, there were 21 m^6^A(N^6^-methyladenosine) modification sites of HBV transcripts in PLC/PRF/5 cells (Table 1) and 20 in Huh7 cells (Table 4), which were the same except that there was one more 512 nt site in PLC/PRF/5 cells; there were seven m^5^C(5-Methylcytosine) modification sites of HBV transcripts in PLC/PRF/5 cells (Table 2) and six in Huh7 cells (Table 5). These m^5^C modification sites shared great agreement in PLC/PRF/5 cells and Huh7 cells, except for the 254 nt site, which was only detected in PLC/PRF/5 cells. Moreover, there were two pseU(Pseudouridine) modification sites of HBV transcripts both identified in PLC/PRF/5 cells and Huh7 cells (Table 3 and Table 6).

Most of the shared m^6^A, m^5^C, and pseU modification sites may be core elements of transcriptional function that had been screened by HBV during evolution. HBV showed a highly conserved cluster of m^6^A modifications (20 loci overlapping) in PLC/PRF/5 (21) versus Huh7 (20) cells. Individual sites of difference between PLC/PRF/5 and Huh7 cells (e.g., m^6^A, m^5^C sites unique to PLC/PRF/5) suggested that HBV responds to host cell epigenetic background heterogeneity may have a dynamic response process. PLC/PRF/5 as an HBV-integrative cell model with HBx-driven reprogramming of histone modifications, such as enrichment of H3K27ac, may up-regulate the activities of modifying enzymes such as METTL3 and NSUN2 [32]. This may give HBV a more efficient replication and immune escape potential in PLC/PRF/5 cells. In contrast, the hepatocellular carcinoma-derived epigenome of Huh7 cells may exert a restrictive effect on the formation of certain modification sites. Additionally, we compared the modification sites across cell lines using classical transcripts, and similarly found that the modification patterns were largely similar across different cells.

**Figure 6:**
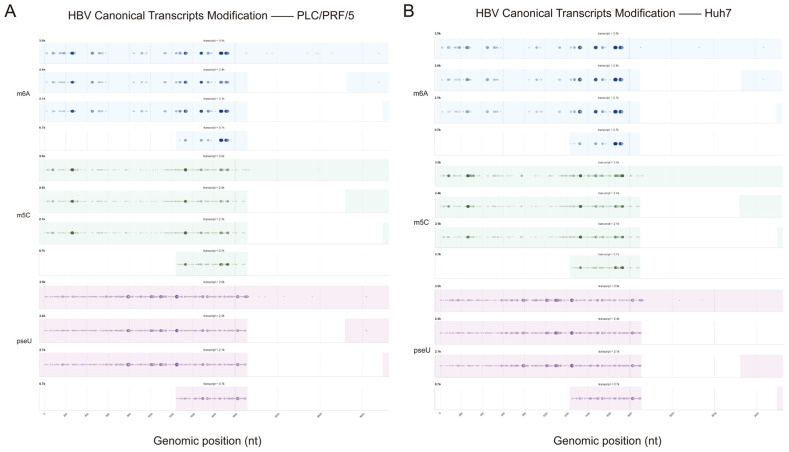
HBV canonical transcript modification site comparison map. (**A**) HBV canonical transcript modification site comparison map in PLC/PRF/5 (m^6^A: blue, m^5^C: green, pseU: purple). (**B**) HBV canonical transcript modification site comparison map in Huh7 (m^6^A: blue, m^5^C: green, pseU: purple). The shape, size, and color of bubbles indicate the degree of modification—larger bubbles with darker colors signify a higher modification rate.

Based on our comparative analysis of modification sites across different cell lines using classical transcripts, we observed that the modification patterns were largely conserved, indicating a consistent post-transcriptional regulatory mechanism for HBV across diverse cellular environments (Figure 5E–H). Furthermore, a comparative study of modification sites on canonical transcripts in two liver cancer cell lines revealed that while the modification sites were largely consistent, their relative abundances exhibited significant differences (Figure 6). Similarly to previous findings, post-transcriptional modification patterns of HBV RNA across different cell lines also exhibit high site-specific conservation. This suggests that HBV likely exploits a highly conserved host epigenomic mechanism to maintain its RNA stability, translation efficiency, and ultimately viral replication—a mechanism applicable across diverse cellular environments. Differences in the relative modification abundance of these sites across two hepatocellular carcinoma cell lines may indicate that while viral RNA modification sites are relatively fixed, the extent of modification may be regulated by cell-type-specific factors (such as the expression levels of methyltransferases/demethylases) [35].

These findings implied that host cells at different stages of liver injury may be guided by differences in modification profiles to switch the ‘immune escape-replication expansion’ survival strategy of HBV to adapt to the challenges of survival in different cellular microenvironments, which laid the foundation for investigating the complexity of HBV gene expression regulation.

## 4. Discussion

Given the widespread global transmission of HBV infection, in-depth research into its life cycle and its effects on host cells is of particular importance [36]. While traditional molecular biology techniques have provided us with basic information about HBV, research into its post-transcriptional processing modifications remains relatively limited. This study investigated post-transcriptional processing modifications of HBV using two distinct hepatocellular carcinoma cell lines: PLC/PRF/5 cells (derived from the primary hepatocellular carcinoma tissue of a 24-year-old African male) harbor an integrally intact HBV genome, while Huh7 cells (derived from the well-differentiated hepatocellular carcinoma tissue of a 57-year-old Japanese male) exhibit high transfection efficiency and transcriptional activity, making them an ideal model for HBV plasmid transfection. Using Nanopore direct RNA sequencing technology, we systematically analyzed alternative splicing, epigenetic modifications, and poly(A) tail characteristics of HBV transcripts, elucidating post-transcriptional processing modifications of HBV in different hepatocellular carcinoma cell lines [37,38].

RNA is a highly structured macromolecule with various post-transcriptional modifications, including 3′ polyadenylation, alternative splicing, and chemical modifications [33]. As with the eukaryotic transcriptome, selective splicing plays a crucial role in the replication cycle of numerous viral families, contributing to the production of infectious proteins through expression. For instance, HIV-1 primary transcripts undergo extensive and complex selective splicing to produce regulatory viral proteins [39]. Similarly, human papillomavirus (HPV) requires constitutive and selective splicing to generate 20 different mRNAs that encode proteins essential for completing its life cycle [40]. In this study, a total of 34 HBV splice variants were identified (20 of which have been reported), 16 of which were shared by the two-cell lineage and 9 of which were unique to PLC/PRF/5 and Huh7, respectively. As in other studies, we found that D2 (461 nt) and D7 (2450 nt) were the major splice donor sites and that A3 (490 nt) and A7 (1386 nt) were the major acceptor sites. The major HBV splice variant was 2447/489 (with shear editing between 2447 and 489 nt), accounting for the majority of spliced transcripts in this study [31]. The nine unique isoforms for each of the two cell lines may suggest that host factors regulate splice site selection. For example, the enrichment of particular isoforms in cells could be linked to the expression of cell-specific splicing factors (e.g., hnRNPs or SR proteins), which control splicing efficiency by binding to exon/intron silencers (ESS/ISS) or enhancers (ESE/ISE) [41].

Polyadenylation is a conserved regulatory process in eukaryotic gene expression: triggered by RNA polymerase II termination, it initiates 3′-end splicing and catalyzes the formation of a poly(A) tail via polyadenylate polymerase (PAP). By coordinating mRNA maturation, nuclear export, stability, and translation initiation, it plays a central role in cellular homeostasis and disease [42]. Previous study found that the length of the poly(A) tail of bovine coronavirus mRNA changes during infection, decreasing from 45 nucleotides (nt) immediately after viral entry to 65 nt at 6–9 h post-infection and increasing to 120–144 nt at 30 h post-infection. This suggests that longer poly(A) tails may favor translation in coronaviruses [43]. In our study, we found that poly(A) tail lengths differed significantly between classical and long HBV transcripts in PLC/PRF/5 and Huh7 cells, indicating the stability of long HBV transcripts differs between the two cell types. Additionally, the average poly(A) tail length of most variable shear transcript species in Huh7 cells was slightly longer than in PLC/PRF/5 cells. This finding may provide new insights into the heterogeneity of viral antigen expression in different hepatocellular carcinoma models.

Since the late 1950s, more than 300 chemical modifications (epitranscriptomes) have been identified, regulating cellular/viral processes and more than 100 human diseases. There are trans-viral studies showing the universality of the biological functions of such conserved clusters: in HIV-1, for example, the m^6^A cluster at the 3′ end was shown to be an essential element for viral replication, and its absence leads to replication defects at the level of the single-molecule epitranscriptome [37]. The m^5^C modification in the Epstein–Barr virus-encoded protein 1 (EBER1) is crucial for viral lytic replication and negatively impacts RNA stability [19]. In our study, 30 (PLC/PRF/5) and 28 (Huh7) HBV modification sites were detected, including conserved m^6^A (20 shared, 95% overlap), m^5^C (6 shared), and Ψ (2 shared) clusters—likely evolutionarily selected core regulatory elements. Such conserved clusters may serve as core functional elements for adaptive selection of HBV during long-term evolution, driving key processes in viral transcriptional regulation. Meanwhile, cell-specific modification sites (e.g., m^6^A-512 nt and m^5^C-254 nt unique to PLC/PRF/5) reflect HBV’s dynamic adaptation to host epigenetic heterogeneity, enabling plasticity in response to liver injury stages, immune pressure, and replication demands.

This study reveals the epigenomic characteristics of hepatitis B virus (HBV), though the findings represent preliminary research. The identified modification sites and splicing variants require further validation. The functional roles of these modifications and splicing variants in influencing HBV biological properties, viral replication, pathogenicity, and carcinogenicity should be elucidated in future studies.

Collectively, our nanopore direct RNA sequencing study achieved four key outcomes: single-molecule analysis enabled precise identification of low-frequency HBV splicing variants; direct detection of natural RNA modifications avoided chemical biases of traditional methylation sequencing; poly(A) tail profiling overcame short-read sequencing limitations; and integrated multidimensional analysis revealed a synergistic regulatory network involving splicing, modifications, and poly(A) tails. Additionally, by comparing differences in post-transcriptional processing modifications of HBV between PLC/PRF/5 and Huh7 cells, we can infer that PLC/PRF/5 cells may better mimic HBV chronic infection immune evasion by restricting viral replication (host-mediated transcriptional silencing) while maintaining HBsAg secretion. In contrast, the long poly(A) tail in Huh7 cells supports efficient replication, making it potentially more suitable for studying HBV transcription and replication. These findings may provide clues for selecting cellular models relevant to HBV mechanism research and lay the groundwork for predicting antiviral targets.

## Figures and Tables

**Table 1:** Sequencing data of HBV plasmid transfected PLC/PRF/5 cells—m6A modification sites.

Chrom	Position	Name	Valid-Coverage	Percent-Modified	Count-Modified
NC_003977.2	82	a	4530	25.23	1953
NC_003977.2	186	a	7545	39.64	3029
NC_003977.2	254	a	7360	53	3922
NC_003977.2	265	a	7481	98.94	7402
NC_003977.2	289	a	6496	23.45	1596
NC_003977.2	452	a	7363	85.67	6566
NC_003977.2	513	a	7295	25.79	1941
NC_003977.2	523	a	7736	37.01	2908
NC_003977.2	547	a	6977	29.52	2077
NC_003977.2	920	a	7066	41.73	2981
NC_003977.2	1141	a	5203	41.44	2193
NC_003977.2	1245	a	7350	40.23	2981
NC_003977.2	1280	a	7007	65.36	4606
NC_003977.2	1332	a	8278	98.05	8178
NC_003977.2	1339	a	6337	60.47	3898
NC_003977.2	1481	a	8271	87.79	7341
NC_003977.2	1546	a	7336	66.06	5052
NC_003977.2	1664	a	7416	96.5	7204
NC_003977.2	1672	a	8050	96.41	7944
NC_003977.2	1716	a	8141	97.57	7987
NC_003977.2	1731	a	7973	70.48	5656

**Table 2:** Sequencing data of HBV plasmid transfected PLC/PRF/5 cells—m5C modification sites.

Chrom	Position	Name	Valid-Coverage	Percent-Modified	Count-Modified
NC_003977.2	83	m	4530	25.23	1953
NC_003977.2	255	m	7545	39.64	3029
NC_003977.2	266	m	7360	53	3922
NC_003977.2	1333	m	7481	98.94	7402
NC_003977.2	1482	m	6496	23.45	1596
NC_003977.2	1665	m	7363	85.67	6566
NC_003977.2	1732	m	7295	25.79	1941

**Table 3:** Sequencing data of HBV plasmid transfected PLC/PRF/5 cells—pseU modification sites.

Chrom	Position	Name	Valid-Coverage	Percent-Modified	Count-Modified
NC_003977.2	1000	u	6565	24.1	1582
NC_003977.2	1910	u	4609	27.82	2339

**Table 4:** Sequencing data of HBV plasmid transfected Huh7 cells—m6A modification sites.

Chrom	Position	Name	Valid-Coverage	Percent-Modified	Count-Modified
NC_003977.2	82	a	2168	22.39	873
NC_003977.2	186	a	3100	35.33	1111
NC_003977.2	254	a	3079	47.39	1468
NC_003977.2	265	a	3102	98.81	3065
NC_003977.2	289	a	3084	19.24	628
NC_003977.2	452	a	3422	83.84	2989
NC_003977.2	523	a	4274	30.54	1330
NC_003977.2	547	a	4070	24.37	1002
NC_003977.2	920	a	4430	38.23	1714
NC_003977.2	1141	a	4009	33.31	1364
NC_003977.2	1245	a	5449	34.25	1884
NC_003977.2	1280	a	5945	49.34	2955
NC_003977.2	1332	a	7494	90.76	6857
NC_003977.2	1339	a	6191	38.7	2460
NC_003977.2	1481	a	7300	79.71	5890
NC_003977.2	1546	a	6736	55.33	3916
NC_003977.2	1664	a	6823	94.55	6495
NC_003977.2	1672	a	7254	95.36	7082
NC_003977.2	1716	a	7446	95.38	7142
NC_003977.2	1731	a	7172	69.39	5010

**Table 5:** Sequencing data of HBV plasmid transfected Huh7 cells—m5C modification sites.

Chrom	Position	Name	Valid-Coverage	Percent-Modified	Count-Modified
NC_003977.2	1333	m	4649	58.08	2700
NC_003977.2	1482	m	3080	22.11	681
NC_003977.2	1665	m	2846	43.82	1247
NC_003977.2	1673	m	2491	22.56	562
NC_003977.2	1732	m	4628	42.96	1988

**Table 6:** Sequencing data of HBV plasmid transfected Huh7 cells—pseU modification sites.

Chrom	Position	Name	Valid-Coverage	Percent-Modified	Count-Modified
NC_003977.2	1000	u	6565	24.1	1582
NC_003977.2	1910	u	4609	27.82	2339

## Data Availability

The data supporting the findings of this study are openly available in the NCBI BioProject database under accession number PRJNA1336674 (http://www.ncbi.nlm.nih.gov/bioproject/1336674, accessed on 30 September 2025).

## Supplementary Materials

The following supporting information can be downloaded at https://doi.org/10.5281/zenodo.17453921; Table S1: HBV in vitro transcription primer.

## Abbreviations

The following abbreviations are used in this manuscript:

HBV	hepatitis B virus
HCC	hepatocellular carcinoma
ORFs	open reading frames
cccDNA	covalently closed circular DNA
DRS	nanopore direct RNA sequencing
m^6^A	N^6^-methyladenosine
m^5^C	5-Methylcytosine
pseU	Pseudouridine
SD	splice donor
SA	splice acceptor
